# (2-Methyl-3-nitro­phen­yl)methanol

**DOI:** 10.1107/S160053680902707X

**Published:** 2009-07-18

**Authors:** Jian-hong Zhang, You-sheng Chen, Xi Wang

**Affiliations:** a Department of Pharmacy, Guangdong Food and Drug Vocational College, Guangzhou 510520, People’s Republic of China

## Abstract

The asymmetric unit of the title compound, C_8_H_9_NO_3_, contains two crystallographically independent mol­ecules, whose aromatic rings are oriented at a dihedral angle of 83.29 (3)°. In the crystal structure, inter­molecular O—H⋯O hydrogen bonds link the mol­ecules into chains.

## Related literature

The title compound is an intermediate in the synthesis of the monomer 2-methyl-3-nitrobenzaldehyde, utilized to synthesize ergoline derivatives which have potential use in the treatment of Parkinson’s disease, see: Kozikowski *et al.* (1980 ▶). For a related structure, see: Wu *et al.* (1994 ▶). For bond-length data, see: Allen *et al.* (1987 ▶).
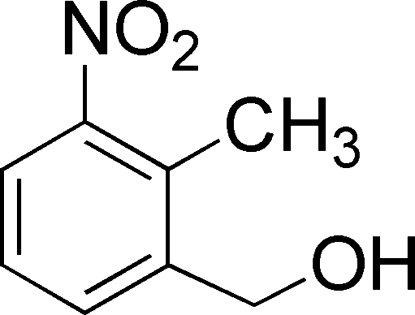


## Experimental

### 

#### Crystal data

C_8_H_9_NO_3_
*M* *_r_* = 167.16
Monoclinic, 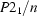

*a* = 13.601 (3) Å
*b* = 7.8650 (16) Å
*c* = 15.433 (3) Å
β = 92.73 (3)°
*V* = 1649.0 (6) Å^3^
*Z* = 8
Mo *K*α radiation
μ = 0.10 mm^−1^
*T* = 294 K
0.30 × 0.20 × 0.10 mm

#### Data collection

Enraf–Nonius CAD-4 diffractometer
Absorption correction: ψ scan (North *et al.*, 1968 ▶) *T*_min_ = 0.969, *T*_max_ = 0.990
3123 measured reflections
2990 independent reflections
1783 reflections with *I* > 2σ(*I*)
*R*_int_ = 0.020
3 standard reflections frequency: 120 min intensity decay: 1%

#### Refinement

*R*[*F* ^2^ > 2σ(*F* ^2^)] = 0.061
*wR*(*F* ^2^) = 0.189
*S* = 1.00
2990 reflections
217 parameters
H-atom parameters constrained
Δρ_max_ = 0.29 e Å^−3^
Δρ_min_ = −0.31 e Å^−3^

### 

Data collection: *CAD-4 Software* (Enraf–Nonius, 1985 ▶); cell refinement: *CAD-4 Software*; data reduction: *XCAD4* (Harms & Wocadlo, 1995 ▶); program(s) used to solve structure: *SHELXS97* (Sheldrick, 2008 ▶); program(s) used to refine structure: *SHELXL97* (Sheldrick, 2008 ▶); molecular graphics: *ORTEP-3 for Windows* (Farrugia, 1997 ▶) and *PLATON* (Spek, 2009 ▶); software used to prepare material for publication: *SHELXTL* (Sheldrick, 2008 ▶).

## Supplementary Material

Crystal structure: contains datablocks I, global. DOI: 10.1107/S160053680902707X/hk2709sup1.cif

Structure factors: contains datablocks I. DOI: 10.1107/S160053680902707X/hk2709Isup2.hkl

Additional supplementary materials:  crystallographic information; 3D view; checkCIF report

## Figures and Tables

**Table 1:** Hydrogen-bond geometry (Å, °)

*D*—H⋯*A*	*D*—H	H⋯*A*	*D*⋯*A*	*D*—H⋯*A*
O1—H1*A*⋯O4^i^	0.82	1.97	2.725 (3)	153
O4—H4*B*⋯O1^ii^	0.82	1.95	2.706 (3)	153

Symmetry codes: (i) 
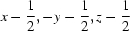
; (ii) 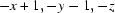.

**Fig. 1.**
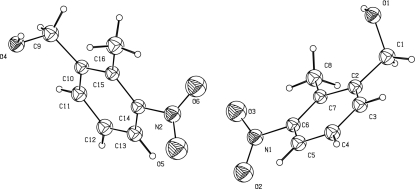
The molecular structure of the title molecule, with the atom-numbering scheme.

**Fig. 2.**
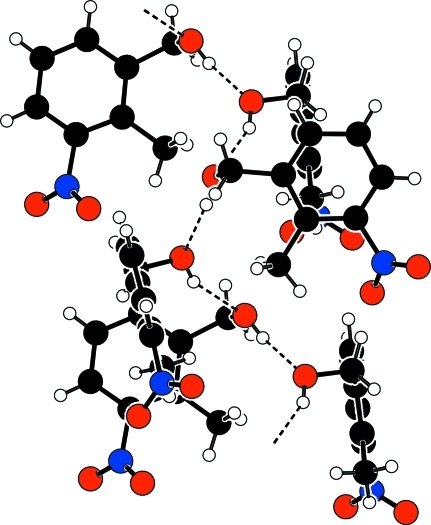
A partial packing diagram of the title compound. Hydrogen bonds are shown as dashed lines.

## supplementary crystallographic information

### Comment

The tittle compound is an important intermediate used to synthesize the monomer 2-methyl-3-nitrobenzaldehyde, which can be utilized to synthesize ergoline derivatives and it has been reported to have some useful physiological and pharmacological functions to parkinson's disease (Kozikowski *et al.*, 1980). We report herein the crystal structure of the title compound, which is of interest to us in the field.

The asymmetric unit of the title compound contains two crystallographically independent molecules (Fig. 1), in which the bond lengths (Allen *et al.*, 1987) and angles are within normal ranges. Rings A (C2-C7) and B (C10-C15) are, of course, planar. The dihedral angle between them is 83.29 (3)°. Atoms N1, C1, C8 and N2, C9, C16 are -0.006 (3), 0.029 (3), -0.022 (3) and 0.077 (3), -0.075 (3), -0.076 (3) Å away from the adjacent ring planes, respectively.

In the crystal structure, intermolecular O-H···O hydrogen bonds (Table 1) link the molecules into chains (Fig. 2), in which they may be effective in the stabilization of the structure.

### Experimental

The title compound was prepared according to a literature method (Wu *et al.*,  1994). Crystals suitable for X-ray analysis were obtained by slow evaporation of methanol for about 20 d.

### Refinement

H atoms were positioned geometrically, with O-H = 0.82 Å (for OH) and C-H = 0.93, 0.97 and 0.96 Å for aromatic, methylene and methyl H, respectively, and constrained to ride on their parent atoms, with U_iso_(H) = xU_eq_(C,O), where x = 1.2 for aromatic and methylene H and x = 1.5 for all other H atoms.

### Crystal data

**Table:**

C_8_H_9_NO_3_	*F*(000) = 704
*M**_r_* = 167.16	*D*_x_ = 1.347 Mg m^−^^3^
Monoclinic, *P*2_1_/*n*	Mo *K*α radiation, λ = 0.71073 Å
Hall symbol: -P 2yn	Cell parameters from 25 reflections
*a* = 13.601 (3) Å	θ = 9–13°
*b* = 7.8650 (16) Å	µ = 0.10 mm^−^^1^
*c* = 15.433 (3) Å	*T* = 294 K
β = 92.73 (3)°	Block, colorless
*V* = 1649.0 (6) Å^3^	0.30 × 0.20 × 0.10 mm
*Z* = 8	

### Data collection

**Table:**

Enraf–Nonius CAD-4 diffractometer	1783 reflections with *I* > 2σ(*I*)
Radiation source: fine-focus sealed tube	*R*_int_ = 0.020
graphite	θ_max_ = 25.3°, θ_min_ = 2.0°
ω/2θ scans	*h* = 0→16
Absorption correction: ψ scan (North *et al.*, 1968)	*k* = 0→9
*T*_min_ = 0.969, *T*_max_ = 0.990	*l* = −18→18
3123 measured reflections	3 standard reflections every 120 min
2990 independent reflections	intensity decay: 1%

### Refinement

**Table:**

Refinement on *F*^2^	Primary atom site location: structure-invariant direct methods
Least-squares matrix: full	Secondary atom site location: difference Fourier map
*R*[*F*^2^ > 2σ(*F*^2^)] = 0.061	Hydrogen site location: inferred from neighbouring sites
*wR*(*F*^2^) = 0.189	H-atom parameters constrained
*S* = 1.00	*w* = 1/[σ^2^(*F*_o_^2^) + (0.098*P*)^2^ + 0.3*P*] where *P* = (*F*_o_^2^ + 2*F*_c_^2^)/3
2990 reflections	(Δ/σ)_max_ < 0.001
217 parameters	Δρ_max_ = 0.29 e Å^−^^3^
0 restraints	Δρ_min_ = −0.31 e Å^−^^3^

### Special details

**Table:**

Geometry. All e.s.d.'s (except the e.s.d. in the dihedral angle between two l.s. planes) are estimated using the full covariance matrix. The cell e.s.d.'s are taken into account individually in the estimation of e.s.d.'s in distances, angles and torsion angles; correlations between e.s.d.'s in cell parameters are only used when they are defined by crystal symmetry. An approximate (isotropic) treatment of cell e.s.d.'s is used for estimating e.s.d.'s involving l.s. planes.
Refinement. Refinement of *F*^2^ against ALL reflections. The weighted *R*-factor *wR* and goodness of fit *S* are based on *F*^2^, conventional *R*-factors *R* are based on *F*, with *F* set to zero for negative *F*^2^. The threshold expression of *F*^2^ > σ(*F*^2^) is used only for calculating *R*-factors(gt) *etc*. and is not relevant to the choice of reflections for refinement. *R*-factors based on *F*^2^ are statistically about twice as large as those based on *F*, and *R*- factors based on ALL data will be even larger.

### Fractional atomic coordinates and isotropic or equivalent isotropic displacement parameters (Å^2^)

**Table:**

	*x*	*y*	*z*	*U*_iso_*/*U*_eq_	
O1	0.16054 (17)	−0.8063 (3)	−0.23189 (13)	0.0674 (6)	
H1A	0.1609	−0.7149	−0.2575	0.101*	
O2	0.1306 (2)	−0.3449 (4)	0.12458 (19)	0.0991 (9)	
O3	0.2061 (3)	−0.2898 (4)	0.0108 (2)	0.1103 (10)	
O4	0.71669 (15)	0.0476 (3)	0.16390 (14)	0.0660 (6)	
H4B	0.7457	−0.0431	0.1706	0.099*	
O5	0.3487 (3)	−0.4236 (4)	0.2226 (2)	0.1256 (13)	
O6	0.4004 (3)	−0.4885 (4)	0.0995 (2)	0.1141 (11)	
N1	0.1592 (2)	−0.3846 (4)	0.0546 (2)	0.0673 (8)	
N2	0.3911 (2)	−0.3892 (4)	0.1578 (2)	0.0730 (8)	
C1	0.0769 (3)	−0.8143 (4)	−0.1800 (2)	0.0688 (9)	
H1B	0.0515	−0.9296	−0.1809	0.083*	
H1C	0.0258	−0.7405	−0.2048	0.083*	
C2	0.1004 (2)	−0.7622 (4)	−0.0875 (2)	0.0537 (8)	
C3	0.1075 (2)	−0.8874 (4)	−0.0244 (2)	0.0617 (8)	
H3A	0.0977	−1.0001	−0.0410	0.074*	
C4	0.1285 (2)	−0.8523 (4)	0.0614 (2)	0.0647 (9)	
H4A	0.1324	−0.9392	0.1023	0.078*	
C5	0.1437 (2)	−0.6856 (4)	0.0859 (2)	0.0606 (8)	
H5A	0.1568	−0.6576	0.1439	0.073*	
C6	0.1391 (2)	−0.5609 (4)	0.0230 (2)	0.0524 (7)	
C7	0.1176 (2)	−0.5903 (4)	−0.06465 (19)	0.0507 (7)	
C8	0.1098 (3)	−0.4514 (4)	−0.1320 (2)	0.0739 (10)	
H8A	0.1235	−0.3436	−0.1049	0.111*	
H8B	0.0445	−0.4502	−0.1584	0.111*	
H8C	0.1565	−0.4720	−0.1756	0.111*	
C9	0.6502 (3)	0.0372 (5)	0.0897 (2)	0.0699 (9)	
H9A	0.6754	−0.0445	0.0492	0.084*	
H9B	0.6471	0.1470	0.0611	0.084*	
C10	0.5492 (2)	−0.0142 (4)	0.11181 (18)	0.0530 (8)	
C11	0.4806 (3)	0.1136 (4)	0.1233 (2)	0.0635 (9)	
H11A	0.4984	0.2258	0.1133	0.076*	
C12	0.3874 (3)	0.0797 (4)	0.1490 (2)	0.0685 (9)	
H12A	0.3434	0.1677	0.1579	0.082*	
C13	0.3602 (2)	−0.0863 (4)	0.1612 (2)	0.0638 (9)	
H13A	0.2974	−0.1124	0.1786	0.077*	
C14	0.4266 (2)	−0.2128 (4)	0.14749 (19)	0.0546 (8)	
C15	0.5225 (2)	−0.1851 (4)	0.12415 (18)	0.0530 (7)	
C16	0.5962 (3)	−0.3269 (5)	0.1152 (3)	0.0820 (11)	
H16A	0.5651	−0.4341	0.1255	0.123*	
H16B	0.6502	−0.3111	0.1568	0.123*	
H16C	0.6203	−0.3257	0.0578	0.123*	

### Atomic displacement parameters (Å^2^)

**Table:**

	*U*^11^	*U*^22^	*U*^33^	*U*^12^	*U*^13^	*U*^23^
O1	0.0856 (16)	0.0542 (13)	0.0632 (13)	0.0045 (12)	0.0132 (12)	0.0046 (10)
O2	0.109 (2)	0.094 (2)	0.095 (2)	−0.0099 (17)	0.0212 (17)	−0.0380 (17)
O3	0.147 (3)	0.0667 (18)	0.119 (2)	−0.0378 (18)	0.025 (2)	−0.0038 (16)
O4	0.0626 (13)	0.0569 (13)	0.0782 (15)	0.0068 (11)	−0.0007 (11)	0.0009 (11)
O5	0.160 (3)	0.108 (3)	0.110 (2)	−0.049 (2)	0.018 (2)	0.0217 (19)
O6	0.147 (3)	0.0529 (16)	0.143 (3)	−0.0101 (16)	0.009 (2)	−0.0303 (17)
N1	0.0624 (17)	0.0586 (18)	0.081 (2)	−0.0018 (15)	0.0003 (15)	−0.0078 (16)
N2	0.079 (2)	0.0530 (18)	0.086 (2)	−0.0051 (15)	−0.0099 (17)	0.0042 (17)
C1	0.070 (2)	0.067 (2)	0.069 (2)	−0.0005 (18)	0.0034 (17)	−0.0058 (18)
C2	0.0487 (16)	0.0512 (18)	0.0615 (18)	0.0024 (14)	0.0054 (14)	0.0001 (15)
C3	0.0614 (19)	0.0427 (17)	0.082 (2)	−0.0028 (15)	0.0089 (17)	0.0032 (16)
C4	0.065 (2)	0.058 (2)	0.071 (2)	0.0007 (16)	0.0051 (17)	0.0141 (17)
C5	0.0591 (18)	0.069 (2)	0.0537 (17)	−0.0004 (17)	−0.0001 (14)	0.0035 (16)
C6	0.0454 (16)	0.0469 (17)	0.0652 (19)	0.0016 (13)	0.0056 (13)	−0.0029 (15)
C7	0.0482 (16)	0.0456 (17)	0.0587 (18)	0.0029 (13)	0.0065 (13)	0.0034 (14)
C8	0.095 (3)	0.057 (2)	0.070 (2)	0.0078 (19)	0.0098 (19)	0.0105 (17)
C9	0.078 (2)	0.072 (2)	0.0598 (19)	−0.0006 (19)	0.0034 (17)	0.0053 (17)
C10	0.0611 (19)	0.0521 (18)	0.0451 (16)	0.0034 (15)	−0.0044 (13)	0.0012 (13)
C11	0.076 (2)	0.0417 (17)	0.071 (2)	0.0028 (16)	−0.0163 (17)	−0.0019 (15)
C12	0.062 (2)	0.051 (2)	0.091 (3)	0.0143 (17)	−0.0128 (18)	−0.0143 (17)
C13	0.0557 (18)	0.060 (2)	0.074 (2)	0.0022 (16)	−0.0072 (15)	−0.0117 (16)
C14	0.066 (2)	0.0386 (16)	0.0583 (18)	0.0020 (15)	−0.0084 (15)	−0.0021 (13)
C15	0.0636 (19)	0.0471 (17)	0.0475 (16)	0.0077 (15)	−0.0061 (13)	−0.0039 (13)
C16	0.079 (2)	0.065 (2)	0.103 (3)	0.0218 (19)	0.005 (2)	−0.008 (2)

### Geometric parameters (Å, °)

**Table:**

O1—C1	1.423 (4)	C6—C7	1.390 (4)
O1—H1A	0.8200	C7—C8	1.508 (4)
O2—N1	1.207 (3)	C8—H8A	0.9600
O3—N1	1.208 (3)	C8—H8B	0.9600
O4—C9	1.427 (4)	C8—H8C	0.9600
O4—H4B	0.8200	C9—C10	1.487 (4)
O5—N2	1.209 (4)	C9—H9A	0.9700
O6—N2	1.203 (4)	C9—H9B	0.9700
N1—C6	1.491 (4)	C10—C11	1.388 (4)
N2—C14	1.480 (4)	C10—C15	1.408 (4)
C1—C2	1.505 (4)	C11—C12	1.372 (5)
C1—H1B	0.9700	C11—H11A	0.9300
C1—H1C	0.9700	C12—C13	1.373 (4)
C2—C3	1.385 (4)	C12—H12A	0.9300
C2—C7	1.414 (4)	C13—C14	1.367 (4)
C3—C4	1.369 (4)	C13—H13A	0.9300
C3—H3A	0.9300	C14—C15	1.387 (4)
C4—C5	1.378 (4)	C15—C16	1.510 (4)
C4—H4A	0.9300	C16—H16A	0.9600
C5—C6	1.379 (4)	C16—H16B	0.9600
C5—H5A	0.9300	C16—H16C	0.9600
			
C1—O1—H1A	109.5	H8A—C8—H8B	109.5
C9—O4—H4B	109.5	C7—C8—H8C	109.5
O2—N1—O3	122.8 (3)	H8A—C8—H8C	109.5
O2—N1—C6	118.2 (3)	H8B—C8—H8C	109.5
O3—N1—C6	119.0 (3)	O4—C9—C10	112.9 (3)
O5—N2—C14	118.0 (3)	O4—C9—H9A	109.0
O6—N2—O5	123.1 (3)	O4—C9—H9B	109.0
O6—N2—C14	118.7 (3)	C10—C9—H9A	109.0
O1—C1—C2	112.5 (3)	C10—C9—H9B	109.0
O1—C1—H1B	109.1	H9A—C9—H9B	107.8
O1—C1—H1C	109.1	C11—C10—C15	119.6 (3)
C2—C1—H1B	109.1	C11—C10—C9	117.8 (3)
C2—C1—H1C	109.1	C15—C10—C9	122.5 (3)
H1B—C1—H1C	107.8	C12—C11—C10	122.1 (3)
C3—C2—C7	120.0 (3)	C12—C11—H11A	118.9
C3—C2—C1	118.5 (3)	C10—C11—H11A	118.9
C7—C2—C1	121.5 (3)	C11—C12—C13	118.9 (3)
C4—C3—C2	122.7 (3)	C11—C12—H12A	120.5
C4—C3—H3A	118.6	C13—C12—H12A	120.5
C2—C3—H3A	118.6	C14—C13—C12	119.1 (3)
C3—C4—C5	118.7 (3)	C14—C13—H13A	120.5
C3—C4—H4A	120.7	C12—C13—H13A	120.5
C5—C4—H4A	120.7	C13—C14—C15	124.2 (3)
C4—C5—C6	118.8 (3)	C13—C14—N2	116.4 (3)
C4—C5—H5A	120.6	C15—C14—N2	119.4 (3)
C6—C5—H5A	120.6	C14—C15—C10	115.9 (3)
C5—C6—C7	124.6 (3)	C14—C15—C16	123.0 (3)
C5—C6—N1	115.4 (3)	C10—C15—C16	121.1 (3)
C7—C6—N1	120.0 (3)	C15—C16—H16A	109.5
C6—C7—C2	115.2 (3)	C15—C16—H16B	109.5
C6—C7—C8	123.7 (3)	H16A—C16—H16B	109.5
C2—C7—C8	121.0 (3)	C15—C16—H16C	109.5
C7—C8—H8A	109.5	H16A—C16—H16C	109.5
C7—C8—H8B	109.5	H16B—C16—H16C	109.5
			
O1—C1—C2—C3	−105.3 (3)	O4—C9—C10—C11	95.5 (3)
O1—C1—C2—C7	73.1 (4)	O4—C9—C10—C15	−82.3 (4)
C7—C2—C3—C4	1.7 (5)	C15—C10—C11—C12	1.9 (5)
C1—C2—C3—C4	−179.9 (3)	C9—C10—C11—C12	−175.9 (3)
C2—C3—C4—C5	−0.4 (5)	C10—C11—C12—C13	−1.9 (5)
C3—C4—C5—C6	−1.3 (5)	C11—C12—C13—C14	0.0 (5)
C4—C5—C6—C7	1.7 (5)	C12—C13—C14—C15	2.1 (5)
C4—C5—C6—N1	−178.4 (3)	C12—C13—C14—N2	−177.2 (3)
O2—N1—C6—C5	−36.1 (4)	O6—N2—C14—C13	126.6 (4)
O3—N1—C6—C5	141.4 (3)	O5—N2—C14—C13	−49.0 (4)
O2—N1—C6—C7	143.8 (3)	O6—N2—C14—C15	−52.8 (4)
O3—N1—C6—C7	−38.7 (4)	O5—N2—C14—C15	131.7 (4)
C5—C6—C7—C2	−0.4 (4)	C13—C14—C15—C10	−2.1 (4)
N1—C6—C7—C2	179.6 (3)	N2—C14—C15—C10	177.2 (3)
C5—C6—C7—C8	177.9 (3)	C13—C14—C15—C16	175.8 (3)
N1—C6—C7—C8	−2.0 (4)	N2—C14—C15—C16	−4.9 (5)
C3—C2—C7—C6	−1.3 (4)	C11—C10—C15—C14	0.1 (4)
C1—C2—C7—C6	−179.6 (3)	C9—C10—C15—C14	177.8 (3)
C3—C2—C7—C8	−179.7 (3)	C11—C10—C15—C16	−177.8 (3)
C1—C2—C7—C8	2.0 (4)	C9—C10—C15—C16	−0.1 (4)

### Hydrogen-bond geometry (Å, °)

**Table:**

*D*—H···*A*	*D*—H	H···*A*	*D*···*A*	*D*—H···*A*
O1—H1A···O4^i^	0.82	1.97	2.725 (3)	153
O4—H4B···O1^ii^	0.82	1.95	2.706 (3)	153

Symmetry codes: (i) *x*−1/2, −*y*−1/2, *z*−1/2; (ii) −*x*+1, −*y*−1, −*z*.
